# Effectiveness of antiviral prophylaxis coupled with contact tracing in reducing the transmission of the influenza A (H1N1-2009): a systematic review

**DOI:** 10.1186/1742-4682-10-4

**Published:** 2013-01-16

**Authors:** Kenji Mizumoto, Hiroshi Nishiura, Taro Yamamoto

**Affiliations:** 1 School of Public Health, The University of Hong Kong, 100 Cyberport Road, Pokfulam, Hong Kong, China; 2 Department of International Health, Nagasaki University Institute of Tropical Medicine and GCOE, Sakamoto, Nagasaki, Japan; 3 PRESTO, Japan Science and Technology Agency, Kawaguchi, Saitama, Japan

## Abstract

**Background:**

During the very early stage of the 2009 pandemic, mass chemoprophylaxis was implemented as part of containment measure. The purposes of the present study were to systematically review the retrospective studies that investigated the effectiveness of antiviral prophylaxis during the 2009 pandemic, and to explicitly estimate the effectiveness by employing a mathematical model.

**Methods:**

A systematic review identified 17 articles that clearly defined the cases and identified exposed individuals based on contact tracing. Analysing a specific school-driven outbreak, we estimated the effectiveness of antiviral prophylaxis using a renewal equation model. Other parameters, including the reproduction number and the effectiveness of antiviral treatment and school closure, were jointly estimated.

**Results:**

Based on the systematic review, median secondary infection risks (SIRs) among exposed individuals with and without prophylaxis were estimated at 2.1% (quartile: 0, 12.2) and 16.6% (quartile: 8.4, 32.4), respectively. A very high heterogeneity in the SIR was identified with an estimated *I*^2^ statistic at 71.8%. From the outbreak data in Madagascar, the effectiveness of mass chemoprophylaxis in reducing secondary transmissions was estimated to range from 92.8% to 95.4% according to different model assumptions and likelihood functions, not varying substantially as compared to other parameters.

**Conclusions:**

Only based on the meta-analysis of retrospective studies with different study designs and exposure settings, it was not feasible to estimate the effectiveness of antiviral prophylaxis in reducing transmission. However, modelling analysis of a single outbreak successfully yielded an estimate of the effectiveness that appeared to be robust to model assumptions. Future studies should fill the data gap that has existed in observational studies and allow mathematical models to be used for the analysis of meta-data.

## Background

In April 2009, a pandemic caused by the influenza A (H1N1-2009) virus (pH1N1) was recognized, spreading rapidly across the world [1]. The virulence of pH1N1 infection appeared to be likely lower than those of other pandemics during the 20th century [2], but the population impact was not negligible as a whole [3]. To strengthen preparedness against future pandemics better with plentiful supports of scientific evidence, it is fruitful to systematically review the lessons from the 2009 pandemic and explicitly analyse the epidemiological dataset [4]. In particular, the effectiveness of containment measure, including contact tracing, case isolation, antiviral prophylaxis, school closure and other radical and resource-consuming countermeasures should be assessed by using the empirical data. This can only be achieved by experiencing an actual pandemic. The datasets from the 2009 pandemic are thus deemed the most valuable source for epidemiological investigations which include an explicit analysis using mathematical and statistical modelling techniques.

Among various public health measures, the present study focuses on the effectiveness of antiviral prophylaxis as part of the containment measure. In the very early stage of the 2009 pandemic, mass chemoprophylaxis was implemented to strictly prevent secondary transmission among close contacts of confirmed cases [5,6]. Since the retrospective epidemiological studies of chemoprophylaxis, commonly coupled with contact tracing, took place across the world, we are granted a precious opportunity to empirically assess the effectiveness of these countermeasures in combination. To date, a number of original studies and systematic reviews have been published on the effectiveness of antiviral prophylaxis against pH1N1, including a study that analysed empirical datasets of pH1N1 in a confined household setting [7]. However, a systematic review has been only partially focused on pH1N1 [8] with a restriction to randomized controlled trials that recruited participants based on some specified definition of “contact”. The estimated effectiveness of prophylaxis derived from such studies has not been directly applicable to other population settings (e.g. not generally applicable to any other contacts) and thus to the associated policymaking. Moreover, a few original studies have rested on very rigorous contact tracing, and the finding has been specific to that particular population (e.g. military conscripts in Singapore [9-11]). Thus, again it is difficult to apply the finding to other practical settings. Similar observational studies have been hardly published.

Given that the effectiveness of chemoprophylaxis at a population level has yet to be explicitly assessed, systematically reviewing and analysing published retrospective observational studies would be a great asset to consider and plan for future contingency planning that involves antiviral prophylaxis. The objectives of the present study are two folds. First, we aim to systematically review the effectiveness of chemoprophylaxis during the course of the 2009 pandemic. Because the effectiveness of chemoprophylaxis in reducing the risk of secondary transmission cannot be separately estimated from that of contact tracing based on retrospective studies, we estimate the effectiveness of the combined two countermeasures. Second, we aim to explicitly estimate the effectiveness of chemoprophylaxis coupled with contact tracing by employing a mathematical modelling technique, focusing on a single epidemic record of a school-driven outbreak.

## Methods

The present study consists of two major analytic steps, i.e. (i) a systematic review of literature and (ii) mathematical modelling of an outbreak data. As for the former, this systematic review was conducted in accordance with the Preferred Reporting Items for Systematic Reviews and Meta-Analyses (PRISMA) statement [12].

### Search strategy

Studies containing data on post exposure chemoprophylaxis against pH1N1 were retrieved from the Medline (PubMed) and Web of Science electronic databases on 2 October 2012. We used the following free text search terms in ‘All fields’:

#1: ‘influenza’ OR ‘flu’ OR ‘H1N1*’ OR ‘pH1N1’ OR ‘nH1N1’ OR ‘vH1N1’

#2: ‘pandemic’

#3: ‘prophylaxis’ OR ‘chemophylolaxis’

#4: ‘antiviral’ OR ‘oseltamivir’ OR ‘zanamivir’ OR ‘neuraminidase’

#5: #1 AND #2 AND #3 AND #4

The search was limited to studies published on or after 20 April 2009, i.e. subsequent to the declaration of emergence of pH1N1 through 2 October 2012.

### Study selection

All titles identified by the search strategy were independently screened by two authors (K.M. and H.N.). Abstracts of potentially relevant titles were then reviewed for eligibility, and selected articles were selected for closer examination if any description of either a complete antiviral chemoprophylaxis or contact tracing was given. The duration of “complete” prophylaxis was defined as those lasting for 7 days or longer. In addition, eligible articles must define the “cases” explicitly and identify exposed individuals based on contact tracing (e.g. based on sharing household or any other opportunities of close contact).

### Ascertainment of secondary cases

Secondary infection risks (SIRs) were calculated as the proportion of identified secondary cases divided by the total number of contacts. It should be noted that the crude calculation of SIRs as an overall conditional risk of infection given exposure involves the following assumptions: (i) all the contacts are equally susceptible, and (ii) the SIR is a conditional risk given exposure to the index case(s), and is examined for a reasonable length of time following an illness onset in the index case. Depending on the case definition, SIRs can take different values due to differential efficiency in identifying pH1N1 infection, e.g. by virologic testing, serologic evidence of infection, a rapid detection testing (RDT) result, or by syndromic definition such as influenza-like illness (ILI) symptoms and acute respiratory infection (ARI). Laboratory methods to confirm pH1N1 infections included the reverse transcription PCR (RT-PCR) or viral culture on specimens collected from the respiratory tract. Serologic methods included an analysis of paired serological specimens by hemagglutination inhibition or microneutralization assays, with a 4-fold or greater rise between baseline and convalescent period conventionally used to indicate an infection. ILI was frequently defined as the presence of fever plus cough or sore throat, i.e., a common surveillance definition of the influenza-like illness. Some studies also reported the occurrence of ARI among contacts where ARI was defined as febrile or afebrile upper respiratory tract infection, commonly in the presence of two or more influenza-related signs or symptoms.

### Data extraction

The primary data extracted were the total numbers of contacts and secondary cases per single primary case with prophylaxis, which were commonly compared against the same data among contacts without prophylaxis. If reported, the information with respect to the number of primary cases with incomplete prophylaxis or who dropped out from the prophylaxis was extracted. Other than these aspects, we extracted the information concerning the ascertainment of secondary cases, the specific outbreak setting (e.g. household or school), the antiviral agents that have been used for prophylaxis, the durations of prophylaxis and other countermeasures, the time from illness onset to starting prophylaxis, containment measures other than contact tracing and chemoprophylaxis, any indication of drug resistance (e.g. report of H275Y mutant), and the numbers of cases with pneumonia and deaths. All the datasets were summarized in a standardized form.

### Statistical analysis of reviewed SIRs

SIRs were stratified according to the presence and absence of prophylaxis. Statistical heterogeneity was assessed by *I*^2^ statistic which represents the extent of the degree of variation. All statistical data were analysed using a statistical software JMP version 9.0.0 (SAS Institute Inc., Cary, NC, USA).

### Modelling method

As a second part of the study, a mathematical model was employed to analyse an outbreak data. We focus on a school-driven outbreak record in Madagascar [13] in which the individual data of the date of illness onset as well as the time periods of mass chemoprophylaxis and school closure have been reported. Because the daily incidence was given, we describe the epidemic dynamics by employing a discrete-time renewal equation model. Let the expected incidence (i.e. the number of new cases) on calendar day *t* be *c*_t_, the linear temporal dynamics of the outbreak is described by

(1) $$c_{t}=\sum_{s=1}^{\infty }A_{s}c_{t-s}\text{,}$$

where *A*_s_ describes the rate of secondary transmission per single primary case at infection-age *s* (i.e., the time since infection) [14,15]. The linear model is employed, because the outbreak occurred in a confined setting and it is unclear if the depletion of susceptible individuals played a role. Thus, as a default assumption, we assume that *A*_s_ is decomposed as

(2) $$A_{s}=Rg_{s}\text{,}$$

where *R* is the reproduction number, representing the average number of secondary cases generated by a single primary case, and *g*_s_ is the probability mass function of the generation time, i.e. the time from infection in a primary case to infection in the secondary case caused by the primary case (see [16] for the details of discretisation). Based on a published statistical study [16], the generation time is assumed to be known and is a discrete function that is derived from the continuous gamma distribution with the mean 2.70 days and the variance 1.21 days^2^. Thus the renewal equation (1) is rewritten as

(3) $$c_{t}=R\sum_{s=1}^{\infty }g_{s}c_{t-s}\text{,}$$

Subsequently, we consider three realistic features of the data-generating process. First, the outbreak investigation study classified the cases into confirmed and non-confirmed cases [13], denoted by *c*_1t_ and *c*_0t_, respectively. While all confirmed cases received antiviral treatment upon diagnosis, non-confirmed cases were not subject to treatment. Supposing that the relative risk of secondary transmission among those with antiviral treatment compared to those without is expressed by a factor (1-*ε*_T_) (i.e. *ε*_T_ is the effectiveness of antiviral treatment in reducing secondary transmissions), the renewal equation should be rewritten as

(4) $$c_{1t}+c_{0t}=\left(1-\varepsilon_{T}\right)R\sum_{s=1}^{\infty }g_{s}c_{1t-s}+R\sum_{s=1}^{\infty }g_{s}c_{0t-s}\text{,}$$

Second, exposed individuals undertook mass chemoprophylaxis with oseltamivir, 75 mg once a day, for 10 days starting from 12 October 2009. Third, the entire school was closed from 16 October until 1 November 2009 to prevent further transmissions at the school. Let *δ*_P_ and *δ*_S_ represent the relative risks of secondary transmission under mass chemoprophylaxis and school closure, respectively. These are dealt with as delta functions, i.e.,

(5) $$\delta_{P}=\{\begin{array}{l} 1,\;\text{for}\;t<\tau_{0}\;\text{and}\;t>\tau_{1}\\ 1-\varepsilon_{P},\;\text{for}\;\tau_{0}\leq t\leq \tau_{1}\; \end{array}$$

and

(6) $$\delta_{S}=\{\begin{array}{l} 1,\;\text{for}\;t<\upsilon_{0}\;\text{and}\;t>\upsilon_{1}\\ 1-\varepsilon_{S},\;\text{for}\;\upsilon_{0}\leq t\leq \upsilon_{1} \end{array}$$

where *τ*_0_ and *τ*_1_ represent the first and last days of antiviral prophylaxis, respectively. Similarly, *υ*_0_ and *υ*_1_ represent the first and last days of school closure, respectively. Parameters *ε*_P_ and *ε*_S_ are the effectiveness of chemoprophylaxis and school closure, respectively. The renewal equation should be updated as

(7) $$E\left(c_{1t}+c_{0t};H_{t-1}\right)=\delta_{P}\delta_{S}R\left[\left(1-\varepsilon_{T}\right)\sum_{s=1}^{\infty }g_{s}c_{1t-s}+\sum_{s=1}^{\infty }g_{s}c_{0t-s}\right]\text{,}$$

in which we denote the history of the series of cases up to day *t* by *H*_t_, and we use the conditional expectation on the left-hand side for the sake of later statistical inference. It should be noted that *R* in (7) is interpreted as the reproduction number in the absence of countermeasures including the chemoprophylaxis, school closure and antiviral treatment. Since the equation (7) describes only the linear dynamics, we also considered an alternative model that explicitly accounts for the depletion of susceptible individuals. The conditional expectation for the alternative model reads

(8) $$E\left(c_{1t}+c_{0t};H_{t-1}\right)=\delta_{P}\delta_{S}Rs_{t}\left[\left(1-\varepsilon_{T}\right)\sum_{s=1}^{\infty }g_{s}c_{1t-s}+\sum_{s=1}^{\infty }g_{s}c_{0t-s}\right]\text{,}$$

where *s*_t_ represents the fraction of susceptible individuals in the beginning of day *t*, which was calculated using the observed data, i.e.,

(9) $$s_{t}=1-\frac{\sum_{s=0}^{t-1}c_{1s}+c_{0s}}{M_{t-1}}\text{,}$$

where *M*_t_ scales the total number of boarders in the beginning of day *t* (i.e. used for scaling the cumulative incidence).

Using the Madagascar data, we estimate four parameters, i.e., *R*, *ε*_T_, *ε*_P_ and *ε*_S_ through a likelihood-based approach. We employ three conditional likelihood functions to estimate the parameters under two different scenarios (i.e. one scenario with the depletion of susceptibles and the other without). As the first likelihood, the infection process is assumed as sufficiently characterized by Poisson process ignoring individual heterogeneity [16]. Given observed data from time 0 up to *t*_f_ with the total daily number of new cases (i.e., the sum of confirmed and non-confirmed cases) denoted by *N*_t_ up to day *t*, the likelihood function is written as

(10) $$L\left(R,\varepsilon_{T},\varepsilon_{P},\varepsilon_{S};H_{t-1}\right)=\prod_{t=1}^{t_{f}}\frac{\text{exp}\left[-E\left(c_{1t}+c_{0t};H_{t-1}\right)\right]E{\left(c_{1t}+c_{0t};H_{t-1}\right)}^{N_{t}}}{N_{t}!}\text{.}$$

Second, an alternative likelihood function is to assume that the incidence is geometrically distributed, which is known to be the case for an exponentially distributed generation time [17].

(11) $$L\left(R,\varepsilon_{T},\varepsilon_{P},\varepsilon_{S};H_{t-1}\right)=\prod_{t=1}^{t_{f}}\frac{1}{E\left(c_{1t}+c_{0t};H_{t-1}\right)+1}{\left(1-\frac{1}{E\left(c_{1t}+c_{0t};H_{t-1}\right)+1}\right)}^{N_{t}}\text{.}$$

Third, if we incorporate a gamma distributed individual heterogeneity for describing the infection process [16], the incidence follows a negative binomial distribution, i.e.,

(12) $$\begin{array}{l} L(R,\varepsilon_{T},\varepsilon_{P},\varepsilon_{S},k;H_{t-1})\\ =\prod_{t=1}^{t_{f}}\frac{\Gamma \left(N_{t}+k\right)}{\Gamma \left(k\right)N_{t}!}{\left(\frac{k}{k+E\left(c_{1t}+c_{0t};H_{t-1}\right)}\right)}^{k}{\left(\frac{E\left(c_{1t}+c_{0t};H_{t-1}\right)}{k+E\left(c_{1t}+c_{0t};H_{t-1}\right)}\right)}^{N_{t}}\text{,} \end{array}$$

where *k* is the dispersion parameter that was estimated jointly with other parameters. Both Poisson and geometric distributions are the special cases of this negative binomial distribution with *k* → ∞ and *k* → 0, respectively. Maximum likelihood estimates of the parameters were obtained by minimizing the negative logarithm of the likelihood function (10), (11) or (12). The 95% confidence interval (CI) was computed by using the profile likelihood. To compare model fits, we employed the Akaike’s Information Criterion.

## Results

### Reviewed literature

**Figure 1:**
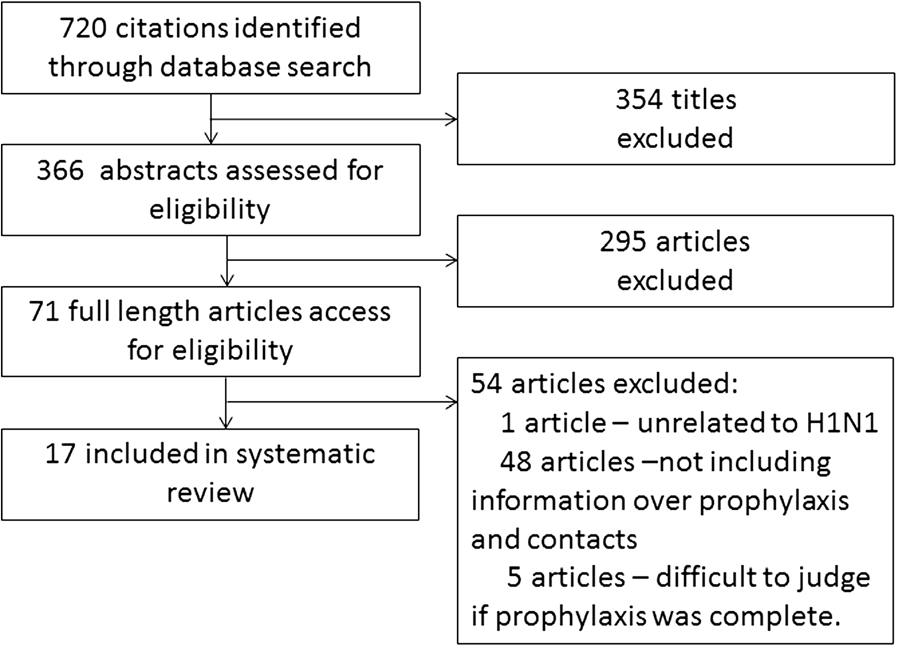
Flow diagram of study selection.

Of the 720 titles that were initially identified, 366 abstracts were accessed for eligibility, of which 295 were excluded, and 71 full length articles were assessed for eligibility (Figure 1). Of these, 17 studies were determined to be eligible and included in this systematic review [6,18-33]. Of the excluded 54 full length reports, 48 articles did not include the information of either prophylaxis or contact, 5 articles did not permit us to differentiate the contacts among those with complete prophylaxis from those with incomplete prophylaxis, and 1 study was on a seasonal A/H1N1. We thus excluded these articles from the following review.

Tables 1, 2, 3 summarize 17 studies that have been included in our review. There was a variety of the definitions of contact, including an arbitrary definition based only on sharing a confined space or an artificial one for the sake of practicalities to disseminate prophylactic medications among all potentially exposed individuals. In the household setting, household contacts tended to be defined as any person who had stayed overnight at least 1 night in the household within 1 day before or 7 days after symptom onset in an index case. In other study settings such as military camp and hospital ward, the definition was broader than household studies, including any person who shared the same space and time with the index case and had a possibility to have been exposed to the index case. Of the 17 studies (which actually covered 19 outbreaks, because one study reported three different outbreaks), 8 (42.1%) reported outbreaks in hospital ward, 9 (47.3%) reported household outbreaks, and 4 (21.1%) outbreaks were school-driven, including those arising from primary school, secondary school and high school (Table 1).

**Table 1:** Studies on contact tracing and antiviral prophylaxis against influenza A (H1N1-2009) included in the systematic review

**Authors**	**Country**	**Year**	**Outbreak Setting**	**Index case ascertainment**	**No. index cases**	**No. subjects traced**	**Postexposure prophylaxis**	**Dose and duration**
Baz et al. [18]	Canada	2009	Household	RT-PCR	1	4	oseltamivir	10 days
Calatayud et al. [19]	United Kingdom	2009	School	RT-PCR	23	2042	oseltamivir	N/A
CDC [20]	Colorado, USA	2009	Long Term Care Facilities	ILI	21	43	oseltamivir	N/A
CDC [20]	Maine, USA	2009	Long Term Care Facilities	ILI/RT-PCR	9	284	oseltamivir	14 days
CDC [20]	New York City, USA	2009	Long Term Care Facilities	RT-PCR	1	983	oseltamivir	N/A
Fallo et al. [21]	Argentina.	2009	Household	RT-PCR	92	266	oseltamivir	N/A
France et al. [22]	USA	2009	School (High School)/Household	ILI/RT-PCR	222	50	oseltamivir (n = 46), zanamivir (n = 4)	N/A
Iioka et al. [23]	Japan	2010	Hospital ward	RDT	1	49	oseltamivir	N/A
Jackson et al. [24]	USA	2009	Household	RT-PCR/Serology	32	9.7^†^	oseltamivir	N/A
Komiya et al. [6]	Japan	2009	Household	RT-PCR	124	333	oseltamivir (n = 232), zanamivir (n = 63) & others (n = 38)	7–10 days
Kute et al. [25]	India	2010	Hospital ward	RT-PCR	1	1	oseltamivir	10 days
Leung et al. [26]	Hong Kong	2009	School (Secondary school)/Household	RT-PCR	65	64	oseltamivir	N/A
Lisena et al. [27]	Italy	2009	Household	RT-PCR	1	5	oseltamivir	10 days
Maltezou et al. [28]	Greece	2011	Hospital ward	N/A	N/A	13	oseltamivir	10 days
Morgan et al. [29]	USA	2009	Household	RT-PCR	N/A	92	oseltamivir	N/A
Pannaraj et al. [30]	USA	2009	Hospital ward	ILI/RDT/RT-PCR	11	21	oseltamivir	9–10 days
Tsagris et al. [31]	Greece	2011	Hospital ward	RT-PCR	2	20	oseltamivir	10 days
van Gemert et al. [32]	Australia	2009	Household	RT-PCR	N/A	57	oseltamivir	N/A
Weston et al. [33]	Australia	2009	School (Primary School)	Not specified	1	83	oseltamivir	N/A

Year, year of study; RDT, rapid diagnostic testing; ILI, influenza-like illness (fever plus cough or sore throat); RT-PCR, reverse transcriptase polymerase chain reaction; N/A, not available; ^†^weighted number, i.e., the figure with decimal point was used in the original study as it was weighted by an inverse of the probability of being sampled to account for oversampling of households with suspected transmission.

**Table 2:** Secondary infection risks (SIR) during contact tracing and chemoprophylaxis against influenza A (H1N1-2009)

**Authors**	**Ages traced**	**No. subjects traced**	**Secondary cases with prophylaxis**	**SIR % (95% CI) with prophylaxis**	**No. w/o prophylaxis**	**Secondary cases w/o prophylaxis**	**SIR % (95% CI) w/o prophylaxis**	**Secondary case ascertainment**
Baz et al. [18]	15, 18, 50 and 59 y/o	4	1	25.0 (0, 67.4)	0	0	NC	RT-PCR
Calatayud et al. [19]	N/A	2042	0	0	N/A	N/A	N/A	RT-PCR
CDC [20]	N/A	43	0	0	N/A	N/A	N/A	ILI
CDC [20]	N/A	284	6	2.1 (0.4, 3.8)	16	0	0	ILI
CDC [20]	N/A	983	176	17.9 (15.5, 20.3)	N/A	N/A	N/A	ILI
Fallo et al. [21]	N/A	266	29	10.9 (7.2, 14.7)	31	12	38.7 (21.6, 55.9)	ARI/ILI
France et al. [22]	Median 45 y/o (range: 0–91 y/o)	50	2	4.0 (0, 9.4)	651	76	11.7 (9.2, 14.1)	ILI
Iioka et al. [23]	N/A	49	6	12.2 (3.1, 21.4)	0	0	NC	RDT/RT-PCR
Jackson et al. [24]	N/A	9.7^†^	3.6^†^	37.1 (6.7, 67.5)	69.3^†^	11.5^†^	16.6 (7.8, 25.4)	Serology
Komiya et al. [6]	Median 43 y/o (quartiles: 0–82)	333	2	0.6 (0, 1.4)	46	12	26.1 (13.4, 38.8)	RT-PCR
Kute et al. [25]	40 y/o	1	0	0	0	0	NC	ILI
Leung et al. [26]	N/A	64	0	0	141	12	8.5 (3.9, 13.1)	RT-PCR
Lisena et al. [27]	Not specified	5	0	0	1	1	100 (NC)	RT-PCR
Maltezou et al. [28]	Median 11 days (0–27 days)	13	0	0	0	0	NC	ILI
Morgan et al. [29]	N/A	92	18	19.6 (11.5, 27.7)	143	12	8.4 (3.8, 12.9)	ARI/ILI/RT-PCR
Pannaraj et al. [30]	2–343 d/o	21	0	0	0	0	NC	ILI
Tsagris et al. [31]	Below 1 y/o	20	1	5.0 (0, 14.6)	0	0	NC	RT-PCR
van Gemert et al. [32]	1–74 y/o	57	1	1.8 (0, 5.2)	65	17	26.2 (15.5, 36.8)	ILI
Weston et al. [33]	N/A	83	2	2.4 (0, 5.7)	0	0	NC	Non Specified

95% CI, 95 percent confidence interval; RDT, rapid diagnostic testing; ILI, influenza-like illness (fever plus cough or sore throat); RT-PCR, reverse transcriptase polymerase chain reaction; N/A, not available; NC, not calculable; ^†^figure with decimal point was used as it was weighted by an inverse of the probability of being sampled to account for oversampling of households with suspected transmission.

**Table 3:** Secondary infection risks (SIR) of influenza A (H1N1-2009) in relation to the timing of prophylaxis, containment measures and other factors

**Authors**	**SIR % (95% CI) with prophylaxis**	**Time to start prophylaxis**	**Containment measures other than contact tracing and chemoprophylaxis**	**Report of H275Y mutant**	**No. of cases with pneumonia**	**No. of deaths**
Baz et al. [18]	25.0 (0, 67.4)	0–2 days	N/A	Yes	N/A	N/A
Calatayud et al. [19]	0	0–10 days	School closure	No	N/A	N/A
CDC [20]	0	0–4 days	Movement to care unit	No	0	0
CDC [20]	2.1 (0.4, 3.8)	N/A	Movement restriction, Facility closure to new admission and visitors	No	N/A	N/A
CDC [20]	17.9 (15.5, 20.3)	within 24 hours	Restriction of visiting	No	N/A	N/A
Fallo et al. [21]	10.9 (7.2, 14.7)	N/A	N/A	No	N/A	N/A
France et al. [22]	4.0 (0, 9.4)	N/A	N/A	No	N/A	N/A
Iioka et al. [23]	12.2 (3.1, 21.4)	immediately after illness onset in index case	Refusal of new admission, Entry restriction	Yes	3 (50%)	3 (50%)
Jackson et al. [24]	37.1 (6.7, 67.5)	N/A	N/A	No	N/A	N/A
Komiya et al. [6]	0.6 (0, 1.4)	median 2 days (quartiles: 0–7)	Staying at home	Yes	N/A	N/A
Kute et al. [25]	0	N/A	N/A	No	0	0
Leung et al. [26]	0	N/A	School closure	No	0	0
Lisena et al. [27]	0	1–2 days	N/A	No	0	0
Maltezou et al. [28]	0	N/A	N/A	No	0	0
Morgan et al. [29]	19.6 (11.5, 27.7)	N/A	N/A	No	N/A	N/A
Pannaraj et al. [30]	0	N/A	N/A	No	0	0
Tsagris et al. [31]	5.0 (0, 14.5)	N/A	N/A	No	1 (100%)	0 (0%)
van Gemert et al. [32]	1.8 (0, 5.2)	median 6 days	Separation and restriction of movement in their homes	No	N/A	N/A
Weston et al. [33]	2.4 (0, 5.7)	1–2 days	Quarantine	No	N/A	N/A

95% CI, 95 percent confidence interval; N/A, not available.

With respect to index case ascertainment (Table 1), RT-PCR was used in 15 outbreaks (78.9%), ILI was adopted in 4 (21.1%) and RDT was used in 2 outbreaks (10.5%), respectively. The numbers of index cases and traced contacts were reported in 16 of 19 (84.2%) and 19 of 19 outbreaks (100%), respectively. The median numbers of index cases and contacts per study were 10.0 (quartile: 1.0, 56.8) and 50.0 (quartile: 13.0, 266.0), respectively. The median number of contacts per index case was calculated at 3.4 (quartile: 1.2, 44.6). As for prophylaxis, 17 outbreaks (89.5%) employed oseltamivir alone. Of the total of 19 outbreaks, 5 (26.3%) outbreaks reported that the duration of prophylaxis was set at 10 days for oseltamivir alone and 7–10 days for a combination of oseltamivir and zanamivir. The different regimens of 9–10 days and 14 days for oseltamivir alone were adopted in 1 (5.3%) outbreak for each.

### Secondary infection risk and prophylaxis

Median SIRs among exposed individuals with and without prophylaxis were estimated at 2.1% (quartile: 0, 12.2) and 16.6% (quartile: 8.4, 32.4), respectively (Table 2). *I*^2^ statistic was calculated at 71.8%. Of the 19 outbreaks, only 8 outbreaks (42.1%) reported SIRs for both of those with and without prophylaxis. Employing a paired *t*-test with n = 8, the SIR among prophylaxis group appeared to be only marginally significantly smaller than that among the control group (p = 0.052). In two studies, the SIRs among those without prophylaxis were smaller than the SIRs among those with prophylaxis, and the underlying reason for this finding was not manually identifiable. SIR among those who undertook prophylaxis was not significantly associated with the number of traced contacts (p = 0.75, a linear regression). Combining the outbreak setting information in Table 1 with those in Table 2, we found that median SIR with prophylaxis at school setting was 1.2% (quartile: 0, 3.6), being smaller than that at non-school settings with 2.1% (quartile: 0, 17.9) (Welch ANOVA, p = 0.03). Outbreaks occurring in households did not yield significantly higher SIR than other settings (p = 0.12, *t*-test). When we stratify the SIR by ascertainment method (i.e. syndromic or laboratory diagnosis), median SIR with prophylaxis ascertained by ILI was 1.9% (quartile: 0, 12.7), which was slightly smaller than those based on RT-PCR with the median SIR of 2.4% (quartile: 0, 18.6). No significant association was identified between SIR and the ascertainment method (p = 0.50, *t*-test).

The median length of delay from exposure to prophylaxis was 1.5 days (quartile: 0.8, 2.5), which was not associated with SIR (p = 0.14, linear regression; Table 3). Median SIR in outbreaks with clearly documented additional countermeasures (e.g. school closure) was 1.8% (quartile: 0, 7.3) which was smaller than other studies with median SIR of 4.5% (quartile: 0, 20.9). However, the difference was not significantly different (p = 0.21, *t*-test). Outbreaks with a report of mutation marker of resistance yielded greater SIR (median 12.2% (quartile: 0.6, 25.0)) as compared to those without any report of resistance (median 1.9% (quartile: 0, 9.4)), but the difference was not significant (p = 0.47, *t*-test). A total of eight studies explicitly documented the numbers of cases with pneumonia and fatal outcome (Table 3). Of these, 1 study reported three deaths due to pneumonia and another study reported one pneumonia case who had eventually recovered.

### Description of the outbreak in Madagascar

**Figure 2:**
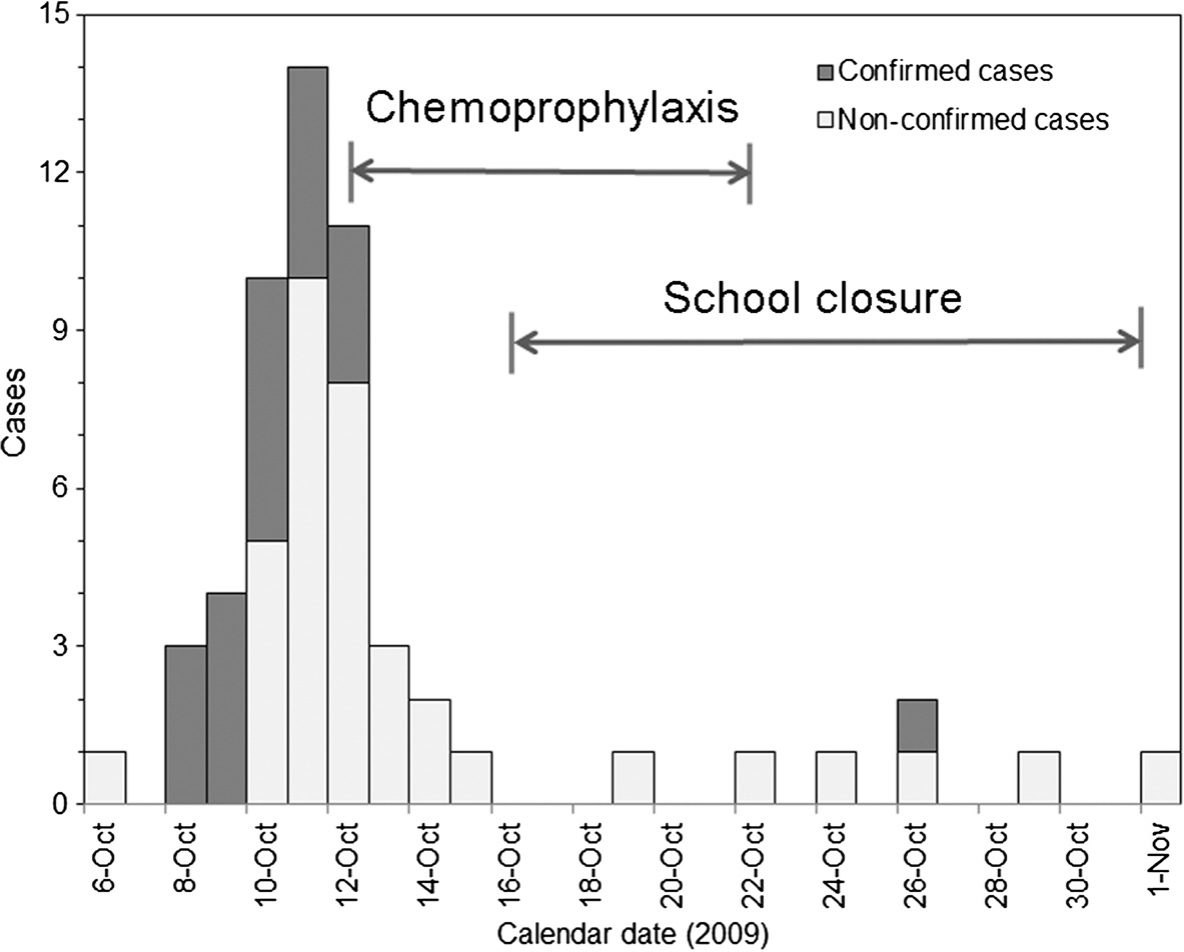
**Epidemic curve of a school-driven outbreak of influenza A (H1N1-2009) in Madagascar. **Daily number of symptomatic cases is shown, depending on confirmatory diagnosis status [13]. Confirmed cases were diagnosed by means of RT-PCR, while others were diagnosed by contact plus influenza-like illness, partially with swab samples. Chemoprophylaxis with oseltamivir was conducted for 10 days from 12 October 2009. School closure was implemented from 16 October 2009.

Figure 2 shows an epidemic curve of a school-driven outbreak of pH1N1 among boarding pupils in Madagascar from 6 October to 2 November 2009. Daily number of new symptomatic cases is shown by the date of illness onset, classified by confirmatory diagnosis status. There were 59 cases who exhibited at least one symptom of influenza-like illness (i.e., fever, cough or sneezing) among a total of 132 boarders. Confirmatory diagnosis was made by real-time RT-PCR. Of the 59 cases, 20 cases (33.9%) were confirmed and 36 cases (61.0%) were non-confirmed in Figure 2. There were other 3 non-confirmed cases (5.1%) who were excluded from Figure 2 and from our modelling analysis due to untraced characters including unknown dates of illness onset. Confirmed cases received antiviral treatment upon diagnosis, while others did not undertake antiviral treatment.

### Modelling results

**Figure 3:**
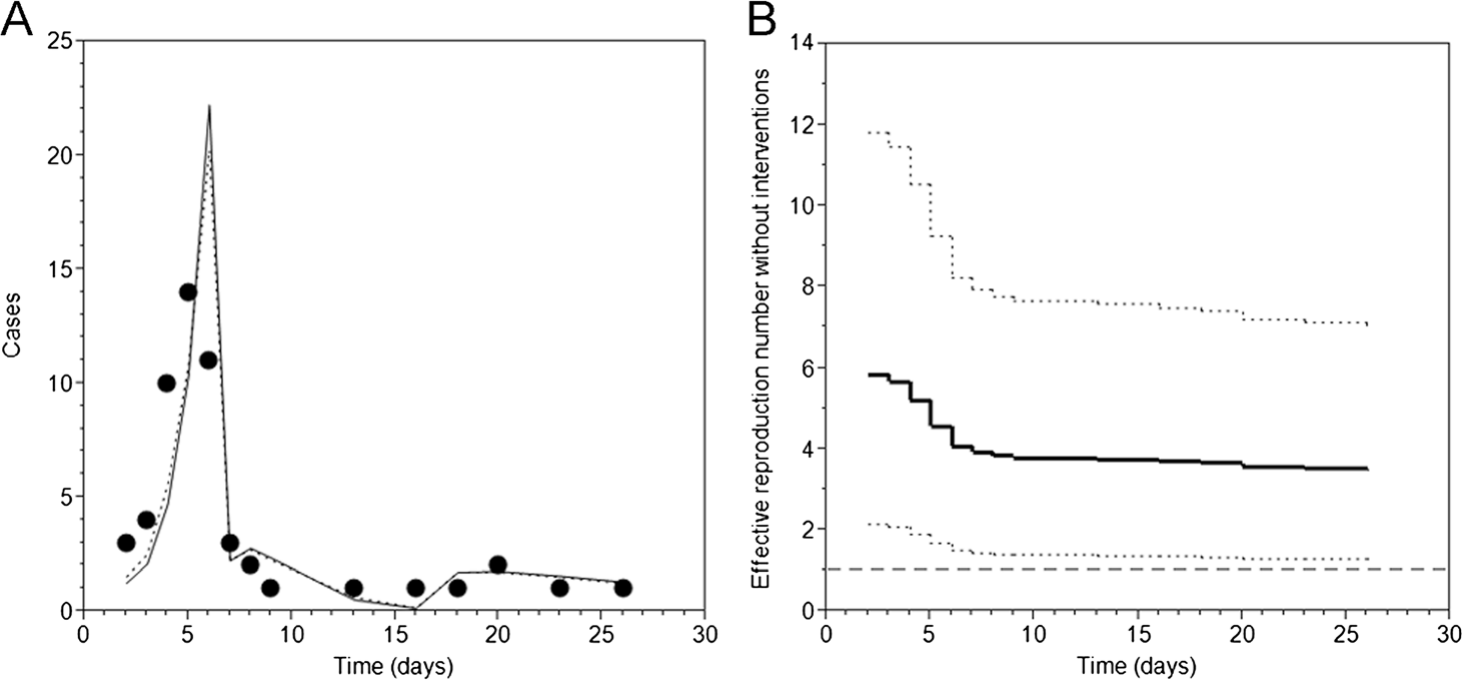
**Epidemic dynamics of influenza A (H1N1-2009) in a primary school, Madagascar. A**. A comparison of the observed daily incidence (dots) against conditional expected values with and without accounting for the depletion of susceptible individuals (solid and dashed lines, respectively). Day 0 represents 6 October 2009 onwards. **B**. Maximum likelihood estimate (solid line) and the 95% confidence intervals (dotted lines) of the effective reproduction number without accounting for interventions. We assumed that the number of cases on each day was sufficiently characterized by Poisson distribution. The estimates of the effective reproduction number reflect the time-dependent reduction in the transmissibility due to depletion of susceptible individuals only. Horizontal dashed line represents the threshold level of *R* = 1 above which it implies that the epidemic can continue without public health interventions.

Figure 3A compares the observed and predicted incidence of pH1N1 outbreak in Madagascar. Overall, our simplistic model captured the qualitative pattern of the temporal dynamics (i.e. incidence) well. Fitting two different types of models with and without accounting for the depletion of susceptibles, we did not find an apparent difference in the fitting results (Figure 3A and Table 4).

**Table 4:** Estimates for the parameters governing the transmission dynamics of influenza A (H1N1-2009) in a primary school in Madagascar, 2009

**Offspring distribution**	**Depletion of susceptibles**	**Reproduction number (95% CI**^**‡**^**)**	**% Reduction in transmissibility by treatment (95% CI**^**‡**^**)**	**% Reduction in transmissibility by contact tracing and chemoprophylaxis (95% CI**^**‡**^**)**	**% Reduction in transmissibility by school closure (95% CI**^**‡**^**)**	**AIC**^**§**^
Poisson	Yes	4.8 (1.8, 9.6)	14.1 (0, 72.1)	94.3 (87.6, 97.7)	31.6 (0, 76.8)	37.9
Poisson	No	6.0 (2.2,12.2)	4.4 (0, 68.2)	93.3 (85.6, 97.3)	10.0 (0, 69.5)	35.0
Geometric	Yes	6.6 (1.5, NC)	26.1 (0, NC)	94.1 (71.8, 98.7)	40.6 (0, NC)	39.7
Geometric	No	7.4 (1.6, NC)	12.8 (NC, NC)	92.8 (66.7, 98.4)	15.1 (0, NC)	39.1
Negative binomial	Yes	6.6 (2.2, 16.4)	27.7 (0, 85.5)	95.4 (87.7, 98.4)	48.0 (0, 84.8)	37.6
Negative binomial	No	7.2 (2.5, 17.0)	13.1 (0, 78.4)	94.0 (85.6, 97.8)	22.6 (0, 76.2)	36.1

^‡^CI, confidence interval (derived from the profile likelihood); ^§^AIC, Akaike’s information criterion; NC, not calculable; Dispersion parameter estimates for negative binomial distribution are 7.6 and 13.0 for those with and without accounting for depletion of susceptibles.

Table 4 summarizes the parameter estimates of the proposed model and the effectiveness of prophylaxis, treatment and school closure. The effectiveness of mass chemoprophylaxis ranged from 92.8% to 95.4% according to different model assumptions and likelihood functions. The estimated high values should be cautiously interpreted as they do not only represent the effect of antiviral prophylaxis but also many other factors that could arise from rigorous contact tracing. The estimated effectiveness of antiviral prophylaxis was less sensitive to the incorporation of the depletion of susceptible individuals and the choice of likelihood function, especially when it was compared with the sensitivities of other parameters including the reproduction number (the MLE ranging from 4.8 to 7.4), the effectiveness of antiviral treatment (the MLE from 4.4% to 26.1%) and the effectiveness of school closure (the MLE ranging from 10.0% to 48.0%). In other words, Table 4 suggests that the effectiveness of chemoprophylaxis coupled with contact tracing that is greater than 92% is a robust finding. With regard to the model fit, the simplest type, i.e., a Poisson distributed likelihood without depletion of susceptibles, yielded the minimum AIC value (35.0).

Figure 3B shows the estimate of the effective reproduction number along with the 95% confidence interval based on a model that explicitly accounted for the depletion of susceptible individuals. Here the effective reproduction number represents the average number of secondary cases per primary case at calendar time *t* in the absence of any interventions. Both the expected value and the lower 95% confidence limit were continuously above unity, indicating that the outbreak could have lasted for a longer time if no intervention took place. In other words, the outbreak was likely to have declined to extinction due to concerted interventions.

## Discussion

The present study explored published literature on antiviral prophylaxis and contact tracing against pH1N1, aiming to systematically investigate the published data and explicitly estimate the effectiveness using a modelling approach that has been already used for exploring various aspects of contact tracing [34-36]. In the systematic review, a very high heterogeneity in the SIR was identified, which was likely associated with differential settings of exposure and non-uniform designs of observational studies. Although the SIR among those who undertook the prophylaxis was significantly smaller than the control group, it appeared infeasible to explicitly estimate the effectiveness of prophylaxis in reducing the transmission of influenza based on a simple meta-analysis of retrospective observational studies. As a supplementary approach, we have devised a mathematical model, applying it to a single particular outbreak in Madagascar and yielding the estimates of the effectiveness of prophylaxis and other countermeasures. A renewal equation model, which has attracted scientific interest in other recent applications [37], was employed. The effectiveness of prophylaxis coupled with contact tracing was estimated as high as 92% or greater, which appeared to be robust to differential model assumptions. This was also in good agreement with the relative reduction in median SIR by prophylaxis, calculated at 87.3%. A mathematical modelling exercise focusing on a specific dataset satisfied the need to explicitly estimate the effectiveness.

There has been a published systematic review of randomized controlled trials (RCT) on the effectiveness of antiviral prophylaxis at an individual level [8]. However, the exposure in the controlled trials has had to be very specific and comparable across studies, and thus, the result has not been directly applicable to a variety of practical settings (e.g. for policymaking at a population level). In fact, there has not been a generally accepted mathematical procedure in translating the individual estimate during a certain contact into the likely effectiveness at a population level (e.g. in a school). This indicates that the population benefit of implementing prophylaxis as part of containment measures has remained unclear even after the RCT. In such an instance, we believe that it is useful to estimate the effectiveness from retrospectively collected data at population levels, even though observational study design is vulnerable to various factors including case ascertainment and indirect impact of contact tracing on the spread of disease. We systematically searched for all potentially available literature, although there was only the limited available information with a difficulty in disentangling the data-generating process. Our review identified that different studies employed different methods of “ascertainment” and different definitions of “exposure”, not allowing us to conduct a simple and explicit meta-analysis. On the other hand, modelling analysis offered a robust estimate of the effectiveness of prophylaxis, demonstrating that the contact tracing with prophylaxis yielded an apparent reduction in the risk of secondary transmission. This finding unfortunately applies only to the specific school setting in Madagascar, but our study adds to literature including studies among military conscripts in Singapore, supporting the notion that it is worth considering the containment measure with antiviral prophylaxis.

An important data gap should be identified for future observational studies, because an explicit statistical analysis could be made based on a well-designed observational study [38]. The designed observational study could also satisfy other objectives including the determination of optimal duration of prophylaxis [39]. As we discussed earlier, one particular study setting offers the estimate that is applicable to only the same setting, preventing us from offering a broadly applicable finding to other populations. However, considering that our modelling exercise successfully estimated the effectiveness of antiviral prophylaxis for a particular outbreak at a school setting, and given that it is difficult to disentangle the relationship between individual effect in RCTs and its relevance to the effectiveness at a confined setting, future study should collect the dataset in a particular confined setting with the details of exposure information (e.g. the time from exposure to prophylaxis and the extent/density of the contact) adopting common methodology for ascertainment and exposure across different studies. One could subsequently employ a mathematical model to analyse the meta-data by combining different datasets of an identical confined setting (e.g. meta-data of school outbreaks). As long as we can ensure the comparability of ascertainment method and contact across different studies during the systematic review, the analysis of meta-data using mathematical modelling techniques could possibly yield more or less comparable and widely applicable results. In fact, a recent modelling study analysed the meta-data of household transmission studies, estimating a key parameter that governs the transmissibility as well as identifying the extent of heterogeneity [40]. Unfortunately, the presently available data on chemoprophylaxis (Table 1) did not permit us to conduct a similar model-based meta-analysis due mainly to inconsistency of the definition of contact and shortage of information. However, future studies can systematically address the abovementioned points by focusing on a specific confined setting such as household or school.

Three technical limitations should be noted. First, our systematic review investigated retrospective observational studies that did not provide us with sufficient epidemiological information and sample size. Because of different outbreak settings with missing data, we did not adhere to formal methodology of meta-analysis and, for instance, did not account for the weight of each study based on sample size when implementing any hypothesis testing. A more controlled analytical method with an identical exposure setting and greater sample size could offer some positive important finding in the future. Second, the outbreak in Madagascar occurred in a school setting and we applied a homogeneously mixing model to the data. The validity of applying such an approximate model to close contact data has yet to be assessed. An explicit validation including the appropriateness of computing the threshold quantity is called for. Moreover, one should remember that our modelling approach ignored asymptomatically infected individuals who could have been infectious to others. Third, the generation time was assumed as known. Although one could try to estimate the generation time jointly with other parameters from epidemic data, the generation time distribution of the small outbreak data should be far from the stable distribution [16] and the time-dependency in a specific population (e.g. boarders) which is likely to form clusters is expected to be complex [41,42].

Despite a need for improvements in a number of different methodological aspects to explicitly assess the feasibility of antiviral prophylaxis as part of containment measure, the present study at least identified the associated epidemiological data gaps. When an outbreak is confined to a particular setting, we have shown that one can use the time of illness onset and the detailed timing of interventions to estimate the effectiveness of prophylaxis by employing a mathematical model. As long as we improve the study designs including common methodologies of ascertainment and contact tracing, mathematical modelling will be a very useful tool to analyse the meta-data and answer pressing public health questions [43].

## Conclusions

The present study systematically reviewed retrospective studies that explored the effectiveness of antiviral prophylaxis during the 2009 pandemic and explicitly estimated the effectiveness by employing a mathematical model. In the systematic review, a very high heterogeneity in the SIR was identified with *I*^2^ statistic at 71.8%. It was difficult to explicitly estimate the effectiveness of prophylaxis based on simple meta-analysis of retrospective observational studies. However, modelling analysis of a single outbreak successfully yielded an estimate of the effectiveness, ranging from 92.8% to 95.4% according to different model assumptions and likelihood functions. Future studies could fill the data gap in retrospective observational studies including the investigation of cases and contacts and analyse the meta-data using a mathematical model.

## Competing interests

The authors declare that they have no competing interests.

## Authors’ contributions

HN conceived the study idea. KM and HN conducted literature review, constructed the epidemiological model and analysed the data. KM and HN jointly drafted the manuscript. YT gave comments and advice on the earlier version of the manuscript. All authors approved the final version of the manuscript.
